# Species characteristics of lead in sea foods collected from coastal water of Fujian, Southeastern of China

**DOI:** 10.1038/srep33294

**Published:** 2016-09-14

**Authors:** Ye He, Zhiqiang Chen, Fan Mo, Limei Huang, LiangJun Xu, Yongning Wu, Zhimin Xue, FengFu Fu

**Affiliations:** 1 Key Laboratory of Analysis and Detection for Food Safety of Ministry of Education, Fujian Provincial Key Lab of Analysis and Detection for Food Safety, Department of Chemistry, Fuzhou University, Fuzhou, Fujian 350116, China; 2 China National Center for Food Safety Risk Assessment, Beijing 100022, China; 3 Fujian Entry-Exit Inspection & Quarantine Bureau, Fuzhou, Fujian 350002, China

## Abstract

Various sea foods including fish, shellfish and shrimp were collected from different coastal areas of Fujian in China, and their Pb species characteristics were investigated in detail. The results indicated that there are two different species characteristics of Pb existing in sea food samples. About half of samples were detected to have only Pb^2+^, and another half of samples were detected to have both Pb^2+^ and trimethyl lead (TML). The results also revealed that Pb species characteristics in the sea foods rather depend on the species of sea food than the sampling area. In comparison with shellfish/shrimp samples, fish samples have higher concentrations of TML and Pb^2+^. Especially, the average concentration of TML in the TML-detected fish samples is about 3 times of that in the TML-detected shellfish/shrimp samples, indicating that fish has stronger ability to uptake and accumulate TML. The concentrations of total lead in all samples are lower than the maximum allowable limit of national standard, suggesting that the sea foods collected from Fujian are safe for consumption. By considering that TAL has more toxicity than Pb^2+^, the effect of TML in sea foods on the human health should be paid more attention in the future.

Lead (Pb) is a non-essential element to the human body, although trace amounts of Pb was considered to be essential to the health of some animals^1^. As a toxic metal, Pb can be accumulated in the human body throughout the lifetime^2^,^3^. It was reported that the excessive intake of Pb will lead to various diseases such as abdominal pain, anaemia, chronic nephritis of the kidney, convulsions, brain damage, central nervous-system disorders and so on^4^,^5^. Therefore, Pb has been classified as a group B2 human carcinogen by the US Environmental Protection Agency (EPA)^6^, and the World Health Organization (WHO) has also established a maximum allowable limit of 10 μg/L for Pb in drinking water^2^. It was reported that the environmental pollution of Pb is primarily due to the manufacture and use of tetra-alkyl lead compounds as petrol additives^7^, thus, there are various Pb species existing in environment. The chemical form of Pb not only dominates its bioavailability and toxicity but also controls its mobility and persistence^7^. Many studies have demonstrated that organolead compounds are more toxic in comparison with inorganic lead (Pb^2+^), and both of organolead and inorganic lead compounds may be accumulated in marine animals and can therefore reach the food chain of human^8^,^9^. For reasons above mentioned, it is crucial to carry out the speciation analysis of Pb in sea foods and further to investigate the species characteristic of Pb in sea foods, in order to evaluate the safety of sea foods from Pb contamination more scientifically.

So far, the Pb levels in some foods such as crops, fruits and vegetables have been investigated by some researchers^10^,^11^,^12^,^13^,^14^,^15^,^16^,^17^,^18^. However, the previous works only determined the total Pb levels in the foods, and the investigation on the species characteristic of Pb in sea foods have been seldom reported due to the difficulty of speciation analysis of Pb^8^. In order to more accurately evaluate the toxicity of lead in foods and further study the mechanisms of lead toxicity, to date, several techniques have been developed for the speciation analysis of Pb based on the combination of separation technology and sensitive element-selective detectors. For example, high performance liquid chromatography (HPLC) and gas chromatography (GC) coupled with atomic emission spectrometry (AES)^19^, mass spectrometry and inductively coupled plasma mass spectrometry (ICP–MS)^20^,^21^,^22^,^23^,^24^,^25^. Not long ago, we developed a environment-friendly microwave-assisted extraction method for quantitatively extracting trace lead compounds from sea foods without altering the individual lead species, and we also developed a sensitive method for simultaneously determining Pb^2+^, trimethyl lead (TML) and triethyl lead (TEL) in sea foods with CE-ICP-MS^26^. The previous research provided a realistic approach for investigating the species characteristic of Pb in sea foods. In this study, we collected different sea foods samples including fish, shellfish and shrimp from different coastal areas in Fujian province, southeastern of China, and investigated the species characteristic of Pb in different sea foods in detail, in order to more accurately and scientifically evaluate the safety of sea foods from lead contamination.

## Results and Discussion

### Confirmation of CE-ICP-MS method for the analysis of Pb^2+^, TML and TEL in sea foods

**Figure 1:**
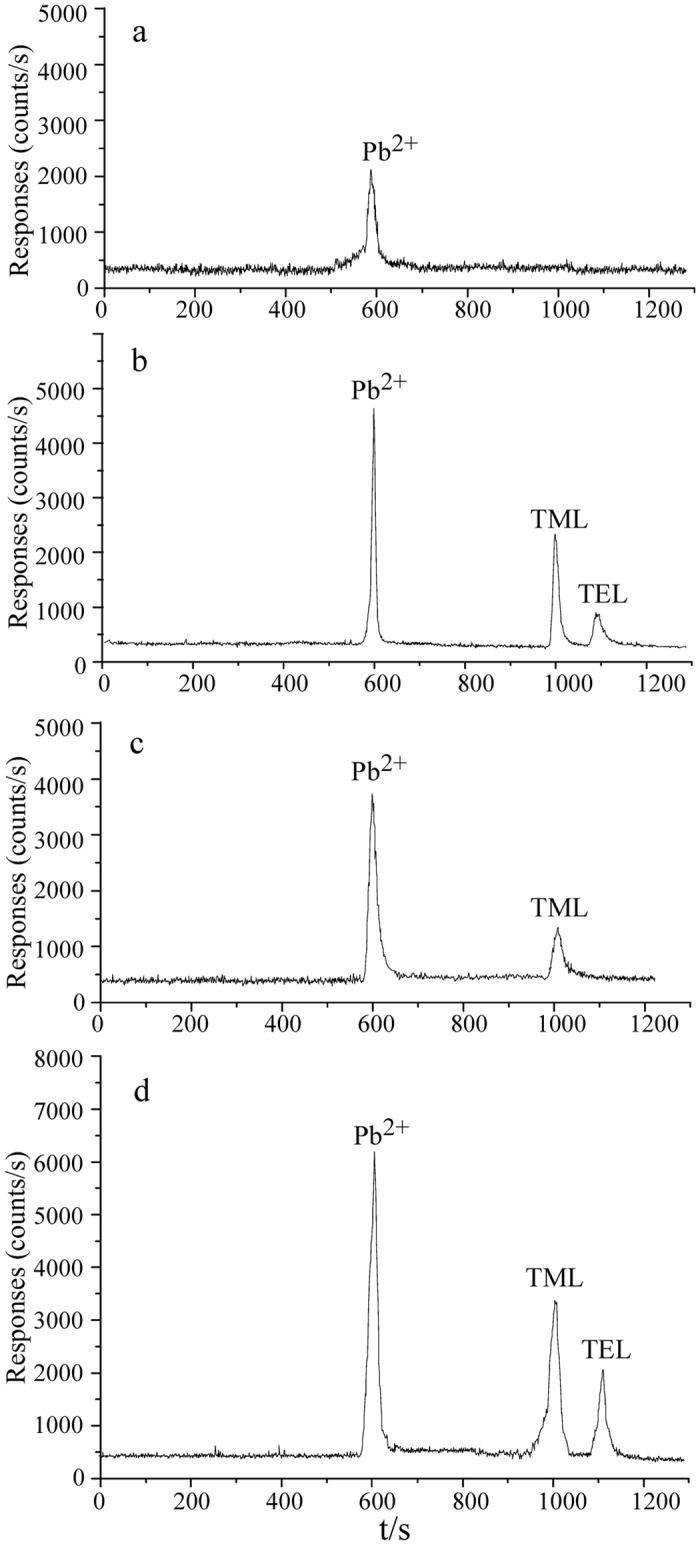
The electropherograms of marine animals under the optimal CE-ICP-MS conditions shown in Table S1. (**a**) Clam (*Paphia undulate*); (**b**) Clam (*Paphia undulate*) spiked with 2 μg/g Pb^2+^, TEL and 0.5 μg/g TML; (**c**) Mandarin fish; (**d**) Mandarin fish spiked with 2 μg/g Pb^2+^, TEL and 0.5 μg/g TML.

To the speciation analysis of lead in sea foods, all Pb species in sea foods samples were completely extracted and each Pb species keep no altering during extraction/analysis process are two key points. As we mentioned above, the microwave-assisted extraction reported in our previous paper were used to extract all Pb species from sea foods samples, and the CE-ICP-MS method reported in the same paper were used for determining Pb^2+^, TML and TEL in this study^26^. In order to confirm the reliability of our methods, the Pb^2+^, TML and TEL in the clam sample (*Paphia undulate*), the mandarin fish sample and a certified reference shrimp material (GBW10050) were extracted and determined with the previous method^26^. The analytical results were shown in Table 1 and their electropherograms were shown in Fig. 1. From Fig. 1, it was clearly observed that Pb^2+^, TML and TEL were baseline separated and determined within 20 min. From the data shown in Table 1, we found that the method has a recovery of 93–104%, a limit of quantification (IUPAC criterion, the concentration to yield a net signal equal to 10 times the standard deviation of background) of 0.4–2.8 ng Pb/g dried weight and a relative standard deviation (RSD, n = 5) <8% for all three Pb species. The sum of the concentrations of each Pb species was consistent with the total Pb concentration, which was obtained with ICP-MS after sample was completely decomposed with 7 mol/L HNO_3_. All above facts indicated that all Pb species in sea foods samples had been completely extracted out, and each Pb species kept no altering during extracting and analytical process since the recovery of at least one Pb species should excessively deviated from 100% if any Pb species was altered during extracting and analytical process. The analytical result of certified reference shrimp material (GBW 10050) obtained with our method is consistent with the certified values, also verifying the reliability of our methods.

**Figure 2:**
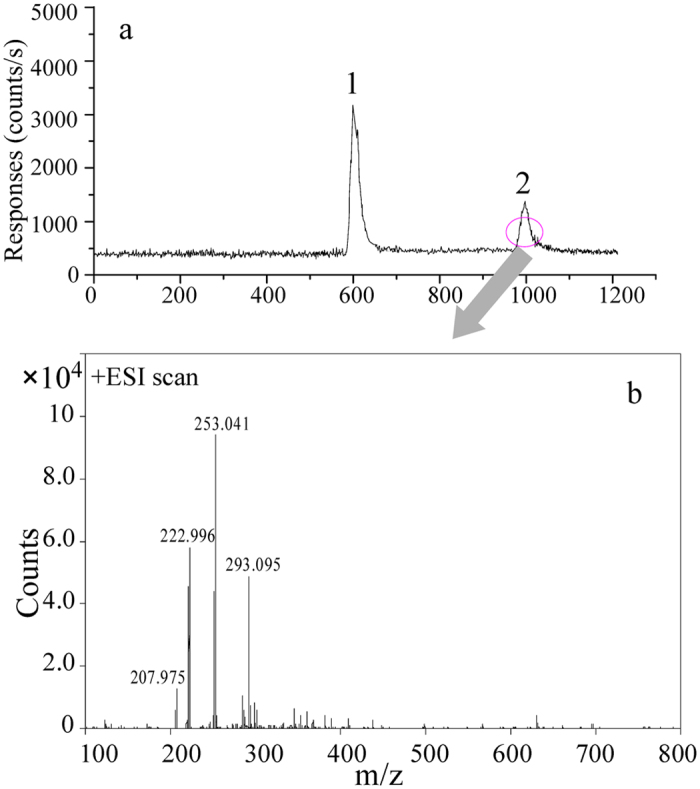
(**a**) The electropherograms of Mandarin fish under the optimal CE-ICP-MS conditions shown in Table S1. (**b**) The ESI-MS results of Mandarin fish. Data was obtained with an Agilent CE-ESI-MS system under Table S1 CE separation condition and ESI-MS conditions as follow: positive mode, 3.5kV electrospray voltage, full scan (m/z from 100 to 800), 0.69 bar of nebulizing gas, drying gas (150 °C) flowed at a rate of 6.0L/mL, the sheath liquid is methanol-water (50:50,v/v) containing 7.5 mM acetic acid.

To further confirm the reliability of our methods, the *Mandarin* fish (tissue) sample, which was detected to have both Pb^2+^ and TAL by CE-ICP-MS, was also determined by CE-ESI-MS (an Agilent ^3D^CE system coupled with an Agilent 1100 series LC/MSD) to identify the existence of TAL. As the data shown in Fig. 2, the ESI-MS results of peak 2 showed a peak at m/z = 253.041, which responding to the positive ion of trimethyl lead, indicating that *Mandarin* fish (tissue) sample really contains TAL and our analytical method is reliable.

### Speciation characteristics of lead in the sea foods collected from Fujian province in south-eastern China

**Figure 3:**
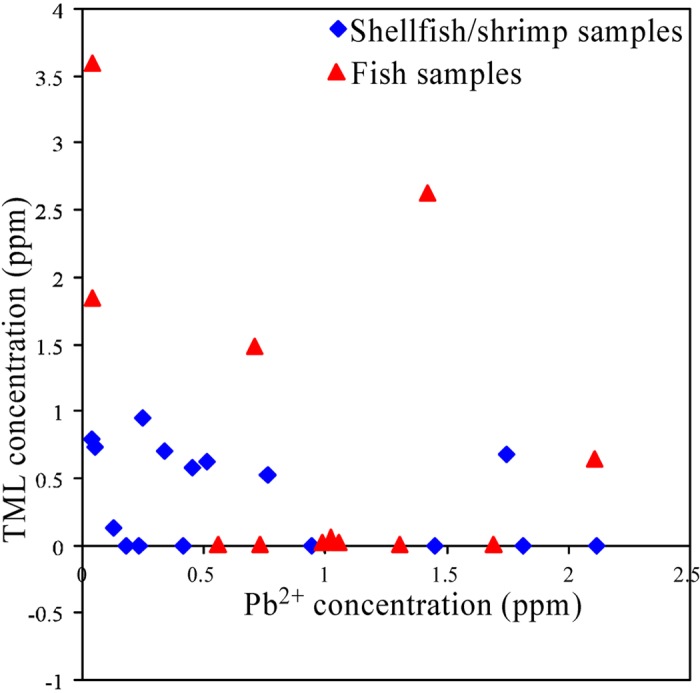
The distributions of Pb^2+^ and TML in all fish samples and shellfish/shrimp samples. Data shown in Tables 2 and 3 were used to plot.

Two typical lead electropherograms of sea foods, obtained with CE-ICP-MS, were showed in Fig. 1, and the analytical results of Pb^2+^, TML and TEL of all sea foods samples were shown in Tables 2 and 3 and Fig. 3. From Figs 1 and 3 and Tables 2 and 3, some features were observed. 1), There are two different lead species characteristics existing in sea foods samples. In some sea foods samples, only inorganic lead (Pb^2+^) was detected; whereas, in other sea foods samples, both Pb^2+^ and TML were detected. In detail, in the case of fish samples, Pb^2+^ was detected in all 12 fish samples, whereas TML was detected in only 5 fish samples. For 16 shellfish/shrimp samples, only Pb^2+^ was detected in 7 samples, and both Pb^2+^ and TML were detected in 9 shellfish/shrimp samples. Totally, TML was detected in about half of sea food samples. 2), Different sea foods, which collected from the same area, showed different species characteristics of lead. For example, *Siniperca chuatsi* (Mandarin fish) and *Pampus sinensis* (Pomfret) collected from Xiamen, and Clam (*Paphia undulate*) and Mussel (*Mytilidae*) collected from Dongshan. As showed in Tables 2 and 3, *Siniperca chuatsi* (Mandarin fish) collected from Xiamen contained both Pb^2+^ and TML, whereas, *Pampus sinensis* (Pomfret) collected from Xiamen contained only Pb^2+^; Clam (*Paphia undulate*) collected from Dongshan contained only Pb^2+^, however, Mussel (*Mytilidae*) collected from Dongshan contained both Pb^2+^ and TML. 3), For the same sea foods, which collected from different area, have the same species characteristics of lead. For example, all *pampus sinensis* (Pomfret), which collected from Xiamen, Longhai, ZhaoAn and Lianjiang, contained only Pb^2+^. As we mentioned above, it was reported that there are various Pb species existing in aqueous environment and the various Pb pollution in the environment mainly come from anthropogenic pollution^7^. So far, the biogeochemical transformation of various Pb species in organism has not been reported. Therefore, undoubtedly, the various Pb species in sea foods are all from their living environment (coastal water). All above facts indicated that the species characteristics of Pb in the sea foods seem rather depend on the species of marine animal than sampling area, although our data are not conclusive owing to the limited number of samples analyzed.

### The concentration distribution of each Pb species in different sea food samples

As we mentioned above, Pb^2+^ was detected in all sea foods samples, whereas, TML was detected in only about half of sea foods samples. From the results shown in Tables 2, 3 and Fig. 3, we can observed that the concentrations of total lead in fish samples is in the range of 0.568–4.298 μg Pb/g dried weight, with a average value of 1.867 μg Pb/g dried weight; and the concentration of total lead in shellfish/shrimp samples is in the range of 0.184–2.606 μg Pb/g dried weight, with a average concentration of 1.143 μg Pb/g dried weight. The Pb^2+^ concentrations in fish samples is in the range of 0.036–2.111 μg Pb/g dried weight, with a average value of 0.971 μg Pb/g dried weight; whereas the Pb^2+^ concentrations in shellfish/shrimp samples is in the range of 0.033–2.115 μg Pb/g dried weight, with a average value of 0.713 μg Pb/g dried weight. For the TML, the fish samples (only for TML-detected samples) have a concentration in the range of 0.645–3.609 μg Pb/g dried weight, with a average concentration of 2.041 μg Pb/g dried weight; whereas the shellfish/shrimp samples (only for TML-detected samples) have a concentration in the range of 0.126–0.954 μg Pb/g dried weight, with a average concentration of 0.633 μg Pb/g dried weight. In comparison with shellfish/shrimp samples, the fish samples not only have a higher concentration of total lead but also have a higher concentration of TML and Pb^2+^. Especially, the average concentration of TML in the TML-detected fish samples is about 3 times of that in the TML-detected shellfish/shrimp samples, indicating that fish has stronger ability to accumulate TML than shellfish and shrimp. The concentration distribution of TAL in TML-detected sea foods also implied that TAL can be more easily up-taken and accumulated by fish than shellfish/shrimp. In 5 TML-detected fish samples, the TML concentration accounted for 68% of total lead averagely; whereas, in 9 TML-detected shellfish/shrimp samples, the TML concentration accounted for only 52% of total lead on average.

As we mentioned above, lead is known to be a toxic metal to human, and the organolead compounds are considered to be more toxic than inorganic lead. In addition, both of inorganic lead and organolead compounds may be accumulated in marine animals and can therefore reach the food chain of human^9^. Therefore, the Chinese Government has established a maximum allowable limit of 0.5 μg/g fresh weight (about 5 μg/g dried weight) for lead in sea foods (GB2762–2005), and the maximum allowable limit of lead in sea foods established by Codex Alimentarius Commission (CAC) is 0.3 μg/g fresh weight (about 3 μg/g dried weight). The concentrations of total lead in all samples detected in this study are lower than the maximum allowable limit of national standard, although the total Pb in 2 fish samples is higher than the maximum allowable limit of CAC, indicating that the sea foods collected from coastal areas of Fujian, Southeastern of China are safe for consumption. Since the maximum allowable limit of TML in sea foods has not been established, therefore, the potential TML risk of sea foods to the consumers can not be discussed in this study. By considering that TAL has more toxicity in comparison with Pb^2+^, the effect of TML in sea foods on the human health should be paid more attention in the future.

## Conclusion

In summary, various sea foods samples including fish, shellfish and shrimp samples were collected from different coastal areas in Fujian province, southeastern of China, and the species characteristics of Pb in them were investigated in detail. The experimental results indicated that about half of sea foods samples were detected to have only Pb^2+^, and another half of sea foods samples were detected to have both Pb^2+^ and TML. The species characteristics of Pb in the sea foods samples seem depend rather on the species of sea foods than sampling area. In comparison with shellfish/shrimp samples, the fish samples not only have a higher concentration of total Pb but also have a higher concentration of TML. Especially, the average concentration of TML in the TML-detected fish samples is about 3 times of that in the TML-detected shellfish/shrimp samples, indicating that fish has much stronger ability to uptake and accumulate TML than shellfish and shrimp. The concentrations of total Pb in all samples are lower than the maximum allowable limit of national standard, suggested that the sea foods collected from coastal areas of Fujian, Southeastern of China are safe for consumption. By considering that TAL has more toxicity than Pb^2+^ and the maximum allowable limit of TML in sea foods has not been established, the effect of TML in sea foods on the human health should be paid more attention in the future.

## Methods

### Sampling site and samples

**Figure 4:**
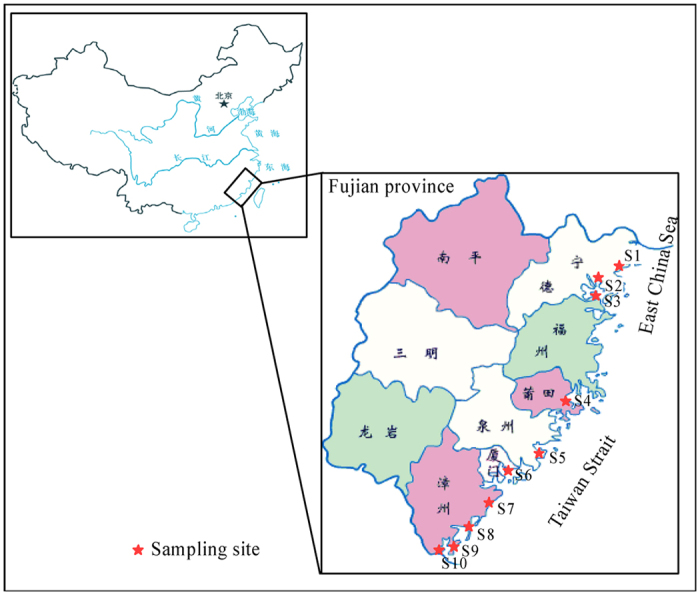
The sampling area of all sea foods, coastal waters of Fujian province in south-eastern Greater China (the map was generated with soft of Canvas 8, Canvas software can be obtained from **http://www.poladigital.co.jp/canvas/index.html**).

About 12 fish samples and 16 shellfish/shrimp samples were collected from different coastal areas of Fujian province in south-eastern China, including XiaPu (S1, 120^o^E, 26.89^o^N), LuoYuan (S2, 119.55^o^E, 26.49^o^N), LianJiang (S3, 119.43^o^E, 26.2^o^N), PuTian (S4, 119.01^o^E, 25.43^o^N), ShiShi (S5, 118.57^o^E, 24.82^o^N), XiaMen (S6, 118.1^o^E, 24.46^o^N), LongHai (S7, 117.78^o^E, 24.36^o^N), ZhangPu (S8, 117.61^o^E, 24.12^o^N), DongShan (S9, 117.4^o^E, 23.72^o^N) and ZhaoAn (S10, 117.16^o^E, 23.63^o^N) (see Fig. 4). All samples were collected in June of 2013, and the details of all samples were shown in Table 4. For each fish sample, 3 fishes were collected at each site, and for each shellfish/shrimp sample, about 1 Kg samples were collected at each site. For fish samples, only muscles were taken and used for analysis. The entire meat of each sample collected from the same site was homogenized and was dried by vacuum freeze drying at −46 °C. The dried meat sample was crushed into powder and was stored in the desiccator for the next speciation analysis of Pb. For shellfish and shrimp samples, the whole tissue was taken by removing their shells, and entire tissue of each sample collected from the same site was homogenized and dried by vacuum freeze drying at −46 °C. The dried tissue sample was crushed into powder and was stored in a desiccator for the next speciation analysis of Pb.

### Speciation analysis of Pb in fish, shellfish and shrimp samples

Each species of Pb in fish, shellfish and shrimp samples was determined by CE-ICP-MS with the method reported in our previous paper^26^. In summary, firstly, inorganic lead (Pb^2+^) and organolead lead including TML and TEL in dried sea foods was separately extracted with a microwave-assisted extraction method by using 50% methanol solution (dissolved in 70 mmol/L H_3_BO_3_–17.5 mmol/L Na_2_B_4_O_7_ solution) and 0.5M acetic acid as solvent respectively. Then, the Pb^2+^, TML and TEL in the extracting solution were determined by CE-ICP-MS under Table S1 conditions (the detailed process was shown in supplementary information).

## Additional Information

**How to cite this article**: He, Y. *et al*. Species characteristics of lead in sea foods collected from coastal water of Fujian, Southeastern of China. *Sci. Rep.* **6**, 33294; doi: 10.1038/srep33294 (2016).

## Supplementary Material

Supplementary Information

## Figures and Tables

**Table 1:** The analytical performance of CE-ICP-MS method for determining Pb^2+^, TML and TEL in different matrix.

			Clam (*Paphia undulate*) tissue				Mandarin fish tissue				GBW10050 (Shrimp)			
aTotal Con.			0.43 ± 0.02				2.45 ± 0.11				0.21 ± 0.01			
	bLOD	cLOQ	dAdded Con.	dDetected Con.	RSD (n = 5, %)	Rec. (%)	dAdded Con.	dDetected Con.	RSD (n = 6, %)	Rec. (%)	dAdded Con.	dDetected Con.	dCertified Con.	Rec. (%)
Pb^2+^	0.12	0.4	0	0.41	6%		0	2.01	6%		0	0.19	0.20 ± 0.05	
			1	1.34	4%	93%	1	3.02	4%	101%	1	1.17		98%
TML	0.41	1.37	0	<DL			0	0.66	6%		0	<DL	ND	
			2	2.06	7%	103%	2	2.51	5%	93%	2	1.87		94%
TEL	0.84	2.80	0	<DL			0	<DL			0	<DL	ND	
			2	1.92	5%	96%	2	1.85	8%	93%	2	2.08		104%

^a^The concentrations determined with ICP-MS after samples were completely decomposed with 7mol/L HNO_3_, unit is μg Pb/g dried weight.

^b^limit of detection (IUPAC criterion, the concentration to yield a net signal equal to 3 times the standard deviation of background), unit is ng Pb/g dried weight;

^c^limit of quantification (IUPAC criterion, the concentration to yield a net signal equal to 10 times the standard deviation of background), unit is ng Pb/g dried weight

^d^Unit is μg Pb/g dried weight.

**Table 2:** Analytical results of each lead species in fish samples collected from Fujian, China.

Sample name	Sampling area	Con. of Pb^2+^ (μg Pb/g dried weight)	Con. of TML (μg Pb/g dried weight)	Con. of TEL (μg Pb/g dried weight)	The Sum Con. (μg Pb/g dried weight)	Total Con. (μg Pb/g dried weight)
*Siniperca chuatsi* (Mandarin fish)	Xiamen	2.111	0.645	—	2.756	2.651
*Pampus sinensis (Pomfret)*	Xiamen	1.304	—	—	1.304	1.325
*Pampus sinensis* (Pomfret)	Longhai	0.998	—	—	0.998	1.002
*Pampus sinensis* (Pomfret)	ZhaoAn	1.030	—	—	1.030	1.051
*Pampus sinensis* (Pomfret)	Lianjiang	1.021	—	—	1.021	1.086
*Epinephelus* (Fish)	Lianjiang	1.689	—	—	1.689	1.702
*Epinephelus* (Fish)	Dongshan	0.729	—	—	0.729	0.744
*Epinephelus* (Fish)	Zhangpu	0.557	—	—	0.557	0.568
*Pagrosomus major* (Fish)	Xiapu	0.710	1.482	—	2.192	2.239
*Decapterus maruadsi* (Fish)	Shishi	0.043	1.845	—	1.888	1.913
*Plectorhynchus cinctus* (Fish)	Luoyuan	1.422	2.622	—	4.044	4.298
*Scomberomorus niphonius* (Pan-Fried Mackerel)	Putian	0.036	3.609	—	3.645	3.936

**Table 3:** Analytical results of each lead species in shellfish and shrimp samples collected from Fujian, China.

Sample name	Sampling area	Con. of Pb^2+^ (μg Pb/g dried weight)	Con. of TML (μg Pb/g dried weight)	Con. of TEL (μg Pb/g dried weight)	The Sum Con. (μg Pb/g dried weight)	Total Con. (μg Pb/g dried weight)
Mussel (*Mytilidae*)	Xiamen	0.033	0.768	—	0.801	0.881
Oyster (*Concha Ostreae*)	Zhangpu	0.178	—	—	0.178	0.184
Abalone	Luoyuan	0.250	0.954	—	1.204	1.235
Clam *(Paphia undulate)*	Zhangpu	0.941	—	—	0.941	1.001
Clam *(Paphia undulate)*	Dongshan	0.418	—	—	0.418	0.426
Mussel (*Mytilidae*)	Dongshan	0.512	0.611	—	1.123	1.218
Mussel (*Mytilidae*)	Zhaoan	0.339	0.698	—	1.319	1.419
Clam *(Ruditapes philippinarum*)	Zhaoan	0.125	0.126	—	0.251	0.242
Clam (*Ruditapes philippinarum*)	Longhai	0.455	0.583	—	1.038	1.109
Prawn	Longhai	1.810	—	—	1.810	1.901
Prawn	Zhaoan	1.452	—	—	1.452	1.493
Razor clam *(Solen grandis)*	Longhai	0.036	0.752	—	0.788	0.797
Clam (*Ruditapes variegates*)	Lianjiang	2.115	—	—	2.115	2.135
Razor clam *(Solen grandis)*	Lianjiang	1.745	0.678	—	2.423	2.606
Razor clam *(Solen grandis)*	Shishi	0.765	0.526	—	1.291	1.407
Clam (*Ruditapes variegates*)	Shishi	0.231	—	—	0.231	0.240

**Table 4:** The sample name and sampling site.

Sample name	Sampling site
Clam *(Ruditapes philippinarum)*	Xiamen (S6), Longhai (S7), ZhaoAn (S10)
Mussel *(Mytilidae)*	Dongshan (S9), ZhaoAn (S10)
Razor clam *(Solen grandis)*	Lianjiang (S3), Shishi (S5), Longhai (S7)
Clam *(Paphia undulate)*	Zhangpu (S8), Dongshan (S9)
Abalone	Luoyuan (S2)
Oyster *(Concha Ostreae)*	Zhangpu (S8)
*Epinephelus* (fish)	Lianjiang (S3), Zhangpu (S8), Dongshan (S9)
*Plectorhynchus cinctus* (fish)	Luoyuan (S2)
*Decapterus maruadsi* (fish)	Shishi (S5)
*Pagrosomus major* (fish)	Xiapu (S1)
*Siniperca chuatsi (*mandarin fish)	Xiamen (S6)
*Pampus sinensis (*pomfret)	Lianjiang (S3), Xiamen (S6), Longhai (S7), ZhaoAn (S10)
*Scomberomorus niphonius* (Pan-Fried Mackerel)	Putian (S4)
Prawn	Longhai (S7), ZhaoAn (S10)
Clam (*Ruditapes variegates*)	Lianjiang (S3)
