# Supplementary material for: Species characteristics of lead in sea foods collected from coastal water of Fujian, Southeastern of China
Source: Sci Rep. 2016 Sep 14;6:33294. doi: 10.1038/srep33294 (PMC5022019; doi:10.1038/srep33294)
Supplement: Supplementary Information [file srep33294-s1.pdf]

Supplementary information for

Species characterization of lead in sea foods collected from coastal water of Fujian, Southeastern China

Ye He[a], Zhiqiang Chen[a], Fan Mo[a], Limei Huang[a], LiangJun Xu[a], Yongning Wu[b], ZhiMin Xue[c] and FengFu Fu[a*]

[a]Key Laboratory of Analysis and Detection for Food Safety of Ministry of Education, Fujian Provincial Key Lab of Analysis and Detection for Food Safety, Department of Chemistry, Fuzhou University, Fuzhou, Fujian 350108,China

[b]China National Center for Food Safety Risk Assessment, Beijing 100022, China

[c]Fujian Entry-Exit Inspection & Quarantine Bureau, Fuzhou, Fujian 350002, China

1. Reagents and apparatus

The standard matters of three species of lead compounds, namely lead chloride (Pb^{2+}), trimethyl lead chloride (TML) and triethyl lead chloride (TEL) were purchased from J&K Co., Ltd. (Beijing, China). The 1000 μg/mL stock standard solution of Pb^{2+} was prepared by dissolving above lead chloride solid in 2% HNO_3, and the 1000 μg/mL stock standard solution of TML and TEL were prepared by dissolving above standard matters in methanol solution. All the stock standard solutions were stored at 5 ºC, and their working standard solutions were prepared by diluting the stock solutions to the desired concentration with Milli-Q water step by step. The super-pure EDTA was obtained from Sangon Biotech Co., Ltd. (Shanghai, China). The super-pure cetyltrimethylammonium bromide (CTAB) was purchased from Sigma Company (USA). The analytical grade of sodium tetraborate ($Na_2B_4O_7 \cdot 10H_2O$) and boric acid (H_3BO_3) were purchased from Shanghai Reagents Co., Ltd. (Shanghai, China). The running buffer solution of 70 mmol/L H_3BO_3-17.5 mmol/L $Na_2B_4O_7$-0.4 mmol/L CTAB-2% methanol (pH 8.85) was prepared by dissolving above regents in Milli-Q water. All solutions were treated by ultrasonic agitation and filtered through a 0.22μm membrane filter before use.

The CE-ICP-MS system, which consisting of a Cei-SP20 CE-Interface system (Reeko instrument Co. Ltd., Xiamen, China) and an Agilent 7500ce ICP-MS (Agilent Technologies, USA), was used for speciation analysis of Pb in this study. The Cei-SP20 CE-Interface system equipped with a 85 cm length×75μm i.d.×375μm o.d. fused silica capillary (Hebei Yongnian Optic Fiber Factory, Hebei, China), and the capillary was conditioned daily by purging with Milli-Q water for 10 min, 0.1 mol/L NaOH solution for 10 min, Milli-Q water for 10 min and running buffer solution for 10min, respectively. After each run, the CE capillary was flushed with Milli-Q water

and running buffer solution for 2 min respectively in order to clear any analyte or matrix adsorbed on the surface of capillary.

All CE-ICP-MS experiments were carried out at room in which the temperature was regulated in 23-25 ºC by an air conditioner, and water used in this experiment is Milli-Q water (18.2 MΩ/cm) prepared by a Milli-Q equipment (Millipore, Bedford, USA).

2. Speciation analysis of Pb in fish, shellfish and shrimp samples

Firstly, all lead species including Pb^{2+}, TML and TEL in dried marine animals was extracted with a microwave-assisted extraction method. Briefly, about 0.5 g dried powder sample was accurately weighed and put into a 30mL Teflon beaker, and then 10 mL of 50 % methanol solution (dissolved in 70 mmol/L H3BO3-17.5 mmol/L $Na_2B_4O_7$ solution) was added into it. The beaker, which closed with screwed cover, was put into a microwave digester (Sineo Microwave Chemical Technology Co. Ltd., Shanghai, China), and the microwave system was programmed to heat the whole at 70 °C for 5 min under 400W power for three times, with a 2 min interval between two heating. After the whole was cooled to room temperature, the extract was separated by filtering it through a 0.22μm membrane filter, and the residues were further extracted with 10 mL of 0.5M acetic acid with the same manner to completely extract inorganic lead. Two extracts were combined into a centrifuge tubes, then, the total extract was evaporated to near dryness by using a pressured nitrogen blowing concentrator with a moderate stream. Finally, the residue was diluted to the appropriate volume with running buffer solution again (according to the lead content in sample), and the final solution was used for the CE-ICP-MS analysis.

For determining Pb^{2+}, TML and TEL with CE-ICP-MS, above 20 μL of above sample solution or mixed standard solution of Pb^{2+}, TML and TEL was firstly put into

a micro tube, and 20 μL of 0.5% EDTA solution was added. Then, the whole was plenty agitated for 10min to complex Pb^{2+} with EDTA. Finally, the whole solution was diluted to 100μL with running buffer solution, and the final solution was injected into CE-ICP-MS for the determination of Pb^{2+}, TML and TEL with electro-migration injection under Table S1 conditions.

Table S1: Optimal Running Parameters of CE-ICP-MS.

Parameters	Value
CE voltage	-12.5 kV
Sample injection time	12 s
CE capillary	i.d. 75 μm; o.d. 365 μm; 85 cm long
Lab Temperature	23 ^{o}C - 25 ^{o}C
Running buffer solution	70 mmol/L H_3BO_3-17.5 mmol/L $Na_2B_4O_7$-0.4 mmol/L CTAB-2 % CH_3OH solution (pH 8.85)
Velocity of pump 1	12 μL/min
Velocity of pump 2	100 μL/min
RF power	1300 W
Outer plasma gas	15 L/min
Intermediate plasma gas	0.90 L/min
Carrier gas	0.75 L/min
Makeup gas	0.30 L/min
Monitored isotope (m/z)	^{208}Pb
Nebulizer type	MCN (optimum flow is 50–200 μL/min)
